# Exercise and fall prevention self-management to reduce mobility-related disability and falls after fall-related lower limb fracture in older people: protocol for the RESTORE (Recovery Exercises and STepping On afteR fracturE) randomised controlled trial

**DOI:** 10.1186/s12877-016-0206-5

**Published:** 2016-02-02

**Authors:** Catherine Sherrington, Nicola Fairhall, Catherine Kirkham, Lindy Clemson, Kirsten Howard, Constance Vogler, Jacqueline CT Close, Anne M Moseley, Ian D Cameron, Jenson Mak, David Sonnabend, Stephen R Lord

**Affiliations:** The George Institute for Global Health, Sydney Medical School, The University of Sydney, Sydney, Australia; Discipline of Occupational Therapy, Faculty of Heath Sciences, The University of Sydney, Sydney, Australia; Sydney School of Public Health, The University of Sydney, Sydney, Australia; Northern Clinical School, Sydney Medical School, The University of Sydney, Sydney, Australia; Department of Aged Care, Royal North Shore Hospital, Sydney, Australia; Prince of Wales Clinical School, University of New South Wales, Sydney, Australia; Neuroscience Research Australia, University of New South Wales, Sydney, Australia; John Walsh Centre for Rehabilitation Research, Sydney Medical School Northern, The University of Sydney, St Leonards, Australia; Department of Geriatric Medicine, Gosford Hospital, Gosford, Australia

**Keywords:** randomised controlled trial, therapeutics, exercise, hip fracture

## Abstract

**Background:**

Lasting disability and further falls are common and costly problems in older people following fall-related lower limb and pelvic fractures. Exercise interventions can improve mobility after fracture and reduce falls in older people, however the optimal approach to rehabilitation after fall-related lower limb and pelvic fracture is unclear. This randomised controlled trial aims to evaluate the effects of an exercise and fall prevention self-management intervention on mobility-related disability and falls in older people following fall-related lower limb or pelvic fracture. Cost-effectiveness of the intervention will also be investigated.

**Methods/Design:**

A randomised controlled trial with concealed allocation, assessor blinding for physical performance tests and intention-to-treat analysis will be conducted. Three hundred and fifty people aged 60 years and over with a fall-related lower limb or pelvic fracture, who are living at home or in a low care residential aged care facility and have completed active rehabilitation, will be recruited. Participants will be randomised to receive a 12-month intervention or usual care. The intervention group will receive ten home visits from a physiotherapist to prescribe an individualised exercise program with motivational interviewing, plus fall prevention education through individualised advice from the physiotherapist or attendance at the group based “Stepping On” program (seven two-hour group sessions). Participants will be followed for a 12-month period. Primary outcome measures will be mobility-related disability and falls. Secondary outcomes will include measures of balance and mobility, falls risk, physical activity, walking aid use, frailty, pain, nutrition, falls efficacy, mood, positive and negative affect, quality of life, assistance required, hospital readmission, and health-system and community-service contact.

**Discussion:**

This study will determine the effect and cost-effectiveness of this exercise self management intervention on mobility-related disability and falls in older people who have recently sustained a fall-related lower limb or pelvic fracture. The results will have implications for the design and implementation of interventions for older people with fall related lower limb fractures. The findings of this study will be disseminated in peer-reviewed journals and through professional and scientific conferences.

**Trial Registration:**

Australian New Zealand Clinical Trials Registry: ACTRN12610000805077.

**Electronic supplementary material:**

The online version of this article (doi:10.1186/s12877-016-0206-5) contains supplementary material, which is available to authorized users.

## Background

Fall-related lower limb and pelvic fractures are common problems with major implications for individuals, their carers, health services and the community. The greatest burden is from hip fractures, and although surgery is generally successful, many people do not fully recover. Most hip fracture survivors do not regain their former levels of activity or mobility and so are at increased risk of further falls. Many also have increased dependence, with approximately 10 % unable to return to their previous residence [1–3]. As the proportion of older rises globally, the costs associated with lower limb and pelvic fractures will increase [4]. Optimising recovery after lower limb fractures and preventing further falls have the potential to reduce the burden on individuals and society. There is systematic review evidence that outcomes after fall-related fractures can be improved with well-designed intervention programs [1, 5–7] but the best approach to improve function is not evident [8]. Current clinical guidelines do not include clear conclusions about the optimal content of rehabilitation programs [9] and systematic reviews provide no consensus on the best type or intensity of exercise after lower limb and pelvic fractures [1, 5, 6].

Existing trials of post-fracture rehabilitation leave many questions unanswered. The interventions that have had the largest effect on mobility and function to date were delivered with high intensity (three times per week) and close supervision in centre-based settings [10, 11]. Such programs are resource intensive and further investigation is indicated to assess whether such gains are possible with less costly interventions. Encouragingly, a recent high-quality randomised trial found a six-month home exercise program, taught by a physiotherapist and undertaken with minimal supervision, improved mobility in older people after hip fracture [12]. This is consistent with previous trials in which home exercise programs taught by physiotherapists improved shorter-term mobility outcomes after hip fracture [13, 14]. Effects on the participation aspect of functioning [15] have not been well investigated to date. Few trials have had intervention periods exceeding six months [16–18] or prolonged follow-up [12]. Considering the evidence for the benefits of ongoing exercise in the older population [19] and de-training once exercise ceases [20], longer intervention and follow-up may be important. Motivational interviewing and self-management approaches have been used in other settings to encourage ongoing intervention adherence but are yet to be well investigated in post hip-fracture populations [21].

Falls remain an important problem in post fracture populations but few trials have evaluated the effect of exercise programs on the prevention of further falls in fracture survivors and the results are conflicting [16, 17, 22]. Bischoff-Ferrari and colleagues found that prescription of home-based exercise by physiotherapists prior to hospital discharge was feasible and that it reduced falls in the following 12 months [17]. Orwig *et al* did not detect a between-group difference in fallers when a home exercise program was compared to usual care [16]. Sherrington *et al* concluded that a home exercise program that did not specifically include fall prevention advice improved mobility (measured with the Short Physical Performance Battery) but actually increased the rate of falls in older people who had recently returned home after hospital stays [22]. There is clearly an urgent need to understand the impact of adding a fall prevention aspect to home exercise programs.

There is potential for a combined home-based exercise and fall-prevention program to be effective and cost-effective in improving mobility and reducing falls following lower limb and pelvic fractures. We have designed such an intervention. The design of this program has been informed by previous research concerning exercise and consumer education for falls prevention and about the role of motivational interviewing and self-management approaches to enhance intervention adherence. Specifically, research suggests that the most effective type of exercise for falls prevention in older people includes a high challenge to balance and a high dose [23], and that the “Stepping On” self-management training program can prevent falls and improve self-efficacy in community-dwelling older people [24].

We will conduct a trial that aims to determine the effect of this exercise and fall prevention self-management training program on mobility-related disability and falls in people with a recent fall-related lower limb or pelvic fracture: the Recovery Exercises and Stepping On after fracture trial (RESTORE) trial. To our knowledge, this is the first study to examine the effect of an exercise and fall prevention self-management program on mobility and falls in older people with a recent lower limb or pelvic fracture. Unlike previous post-fracture studies, the intervention and follow-up will be long-term, and the majority of exercise will be undertaken independently, aiming for a balance between sufficient intensity for effectiveness and reduced cost for feasibility of implementation.

### Primary objective:

To compare the effect of an exercise and fall prevention self-management program with usual care on mobility-related disability and falls in older people with a recent fall-related lower limb or pelvic fracture.

### Secondary objectives

- To compare the effect of an exercise and fall prevention self-management program versus usual care on balance and mobility, falls risk, physical activity, walking aid use, frailty, pain, nutrition, falls efficacy, mood, positive and negative affect, quality of life, assistance from others, hospital readmission, and health-system and community-service contact in older people with a recent fall-related lower limb or pelvic fracture.
- To establish the cost-effectiveness and cost-utility of the intervention approach, compared with that of usual care, from the perspective of the health and community care funder.
- To describe the safety and tolerability of the program.
- To determine features associated with uptake of the intervention.

## Methods / design

### Design

**Fig. 1:**
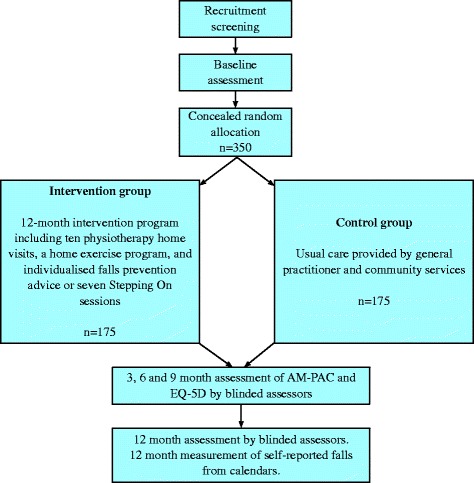
Overview of the flow of participants through the RESTORE trial

A randomised controlled trial will be conducted among 350 people aged at least 60 years with a recent fall-related lower limb or pelvic fracture that led to a hospital admission. Fig. 1 gives an overview of the study design. The Northern Sydney Central Coast Human Research Ethics Committee approved this study (Research Protocol Number 0905-089 M) as did local committees at the recruitment sites. All participants will give written informed consent prior to randomisation. The study adheres to the CONSORT guidelines [25] and is registered with the Australia New Zealand Clinical Trials Register ACTRN12610000805077.

### Participants

Participants will be recruited from Royal North Shore, Prince of Wales, Manly, St George, Mona Vale, Gosford, War Memorial, John Hunter, Royal Newcastle, Longueville Private, Greenwich and Orange Hospitals - all in New South Wales, Australia. Potential participants will be identified in hospital via discussion with hospital staff and review of ward lists. In addition, letters will be sent to potentially eligible people identified from hospital databases, and general advertisement in hospitals, community centres and newspapers will be undertaken.

Participants who meet the following inclusion criteria will be invited to participate: female or male; aged 60 years or older; fall-related lower limb or pelvic fracture in the past two years; no marked cognitive impairment (Mini-Mental State Examination score [26] ≥ 24); living at home or in a hostel (low care residential acre facility where residents generally live in their own units but meals are provided and assistance with showering and dressing in available), in the Sydney, Central Coast, Hunter or Orange regions of New South Wales, Australia.

People will be ineligible to participate in the trial if they meet the exclusion criteria: insufficient English language skills to complete the study assessment and interventions; unable to walk 10 metres despite assistance from a walking aid or another person; progressive neurological disease (e.g., Parkinson’s disease); a medical condition precluding exercise e.g., unstable cardiac disease, uncontrolled hypertension, uncontrolled metabolic diseases, large abdominal aortic aneurysm; currently receiving a treatment program from a rehabilitation facility.

### Randomisation and blinding

After consent and completion of the baseline assessment, participants will be formally entered into the study and randomised to intervention or control groups. Randomisation order will be determined using a computer generated random number schedule with randomly permuted block sizes. Allocation will be concealed by using central randomisation performed by an investigator (CS) not involved in assessments or recruitment, and the treatment allocation tables will be inaccessible to recruitment staff. Staff performing outcome measurement and data analysis for the primary outcomes will be blinded to group allocation. However, due to the nature of the intervention, it is not possible to blind the staff administering interventions or the participants. Participants will be instructed not to inform the assessors of their intervention status, and all exercise equipment will be removed prior to assessment. It is acknowledged that as falls are self-reported, blinding of assessors is not possible for this outcome.

### Intervention

Prior to commencement of the intervention, medical clearance for study participation will be obtained from each participant's medical practitioner.

#### Home exercise program

Participants randomised to the intervention group will each receive 10 home visits and five phone calls from a physiotherapist in the 12-month study period. There will be six visits in the first three months after randomisation and four visits over the following nine months, with each visit lasting 45-60 minutes. Five follow-up phone calls will be made to review progress and address participants’ concerns. The physiotherapist will prescribe an individualised exercise program and use motivational interviewing and goal setting to encourage behaviour change with regard to exercise. Participants will be encouraged to perform the exercise program for 20-30 minutes, three times per week at home, for 12 months. The choice of exercises, degree of difficulty and number of repetitions will be prescribed based upon assessment of the individual participant’s abilities and negotiated with participants. The lower limb strengthening and balance exercises are based on the Weight Bearing Exercise for Better Balance (WEBB) program, developed by investigator CS and colleagues (http://www.webb.org.au). The exercises target strength and control of the lower limb extensor muscles (hip and knee extensors, ankle plantarflexors) with exercises including repetitions of the sit-to-stand movement, semi-squats from a standing position, stepping up onto blocks and heel raises whilst standing on a wedge. Resistance will be applied via body weight, weight-belts or weighted vests as appropriate. Exercises targeting balance will be performed in standing with a progressively narrowed base (feet together, tandem stance, single leg stance), will involve reaching, stepping, and forward, backward and sideways walking. Upper limb support will be minimised in order to adequately challenge balance, but to ensure safety the environment will be set up with stable supports (e.g. bench or table) close by that can be held as necessary. At each visit the physiotherapist will review and adjust the type and intensity of exercises to ensure the intervention remains appropriate and challenging for each participant throughout the study period. Progression will be negotiated with participants. Resistance applied via weight vests or weight belts will start at approximately 2 % of body weight and will be gradually increased so the Borg Rate of Perceived Exertion scale is ‘hard’ (i.e., a rating of 14-16). Maintaining safety while exercising will be a prime consideration when level of difficulty of exercises is prescribed.

The study physiotherapists will be experienced in prescribing exercise for older people. Strategies to optimise adherence to the intervention include goal setting and review at each home visit, scheduling exercise, provision of a home exercise manual that can be updated at each visit by the physiotherapist, and an exercise diary. Further strategies to increase adherence are outlined in Additional file 1. If a participant becomes unwell or is admitted to hospital, the program will be resumed when the participant feels able and the relevant treating professionals deem the participant well enough to participate again.

#### Fall prevention education

Intervention group participants will also be offered the “Stepping On” program [24] (http://www.steppingon.com) as implemented by the New South Wales Ministry of Health (i.e., weekly 2-hour group sessions for seven weeks). The “Stepping On” program is a multi-faceted community based program co-ordinated by an occupational therapist or other health professional in a small-group learning environment. It uses a variety of strategies to increase self-efficacy in fall-risk situations, [27] incorporates a decision-making model to explore barriers and options for reducing risk of falls [28], and uses adult learning principles to self-manage risk [29]. The “Stepping On” content is relevant to people with fall-related lower limb or pelvic fracture and will cover coping with visual loss and regular visual screening, medication management, environmental and behavioural home safety, and community safety [30]. Group sessions will be held in rooms at local community centres or hospitals. Transport will be arranged for those who would otherwise be unable to attend the sessions. Each program will be attended by up to 13 people. The group session will be completed within the 12-month study period, with the timing depending on availability of a “Stepping On” program. The “Stepping On” program encourages the older person to take control and explore different coping behaviours, and encourages follow-through of safety strategies in everyday life. The program addresses each of the eight aspects of the Health Education Impact Questionnaire [31]: positive and active engagement in life, health-directed behaviour, skill and technique acquisition, constructive attitudes and approaches, self-monitoring and insight, health service navigation, social integration and support, and emotional wellbeing.

If participants are unable or unwilling to attend the group-based “Stepping On” program, the physiotherapist will provide them with individualised advice, the weekly “Stepping On” handouts and/or, if deemed appropriate, a publication targeting falls prevention during the scheduled home visits [32]. Verbal advice on fall prevention will be provided to intervention group participants during most home visits.

#### Control group

Participants assigned to the control group will receive the usual care available to older people from their medical practitioners and community services. In the catchment areas for the trial, usual care for non-institutionalised older people may involve allied health input, medical management of health conditions, assessment of care needs, and provision of care. Full documentation of usual care is beyond the scope of this trial.

### Data collection

Data will be collected from medical records (where possible), participant interviews in person and by telephone, physical assessments, and calendars mailed to the research centre. Participants will undergo two home-based assessments; one prior to randomisation and the other 12 months after randomisation. A physiotherapist or a trained research assistant who is unaware of group allocation will perform the assessments. Each assessment will take about one hour to complete.

Details of fracture, comorbidities and hospital stays will be obtained from the medical records where possible. From participant interviews we will obtain baseline information on pre-fracture daily task independence, fracture details, medical conditions, cognition (using the Short Portable Mental Status Questionnaire) [33], nutrition (Mini Nutritional Assessment) [34], medications, and fracture interventions (including surgical fixation, occupational therapy home visits and physiotherapy). The primary and secondary outcomes will be administered at baseline and 12-month assessments.

All participants will receive 12 one-month calendars and questionnaires at the time of the baseline assessment. Participants will be asked to record falls (primary outcome) and use of health and community services on the calendars and to return completed calendars in pre-paid envelopes to the research centre each month. If calendars are not returned, participants will be telephoned to ask about their fall history for that month. Any fall reported on the calendars will be followed up with a phone call to obtain further information about the details and consequences of the fall. All participants will be telephoned at three, six, nine and twelve months for assessment of the Boston University Activity Measure for Post Acute Care (AM-PAC; primary outcome) [35], and European Quality of Life-5 dimensions quality of life assessment (EQ-5D-5 L; secondary outcome) [36]. Staff who receive calendars and questionnaires, make follow up phone calls and enter data will be unaware of group allocation.

### Outcome measures

#### Primary outcome measures

The primary outcomes measured are mobility-related disability and rate of falls. Mobility-related disability will be assessed in three ways: 1) the performance-based Short Physical Performance Battery (SPPB) [37], 2) the self-reported AM-PAC, 3) the self-reported Late Life Functioning and Disability Instrument, Disability Component (LLFDI-DC) [38].

The SPPB examines the ability to stand (for 10 sec) with the feet together in the side-by-side, semi-tandem, and tandem positions; time taken to walk four metres; and time to rise from a chair and return to the seated position five times. The data will be analysed using the lower extremity continuous summary performance score (CSPS) [39]. The activity limitation component of disability will be measured with the computerised version of the AM-PAC, using the basic mobility and daily activity components. The LLFDI-DC will measure the participation restriction element of disability [38].

The number of falls will be assessed using monthly calendars and follow-up telephone calls as required. A fall is defined using the Kellogg definition as an incident where the body unintentionally comes to rest on the ground or another lower level that is not the result of a loss of consciousness, violent blow, or sudden onset of paralysis such as stroke or an epileptic seizure [40].

#### Secondary outcome measures

Secondary outcome measures will be balance and mobility, falls risk, physical activity, walking aid use, frailty, pain, nutrition, falls efficacy, mood, positive and negative affect, quality of life, assistance from others, hospital readmission, and health-system and community-service contact. These measures aim to increase understanding of the effects of the program on the aspects that contribute to an older person’s capabilities and quality of life, and to enable us to conduct economic analyses. The secondary outcomes are described in Table 1. The overall results will be available to participants upon publication of the trial outcomes. Participants will be given their own results if requested; we anticipate participants will express their interest in receiving this information at their 12-month outcome assessment.

**Table 1:** Secondary outcome measures

Domain	Assessment	Description
Balance and mobility	Coordinated stability test [53]	Measures ability to adjust body position in a controlled manner when near the limit of the base of support.
	Maximal balance range test [53]	Measures the maximum distance participants can lean backward and forward.
	Step Test [54]	Dynamic single limb stance is assessed by counting the number of times the participant is able to step one foot on, then off, a 7.5 cm block as quickly as possible in 15 seconds.
	Short Physical Performance Battery (SPPB), individual components	The SPPB components are the ability to stand (for 10 sec) with the feet together in the side-by-side, semi-tandem, and tandem positions; time taken to walk four metres; and time to rise from a chair and return to the seated position five times.
	Choice stepping reaction time [55]	Time to complete a standardised stepping routine onto four white squares on a portable mat, while standing.
Falls and fall risk	Fallers	Proportion of fallers (people having one or more falls) over the 12-month follow-up period.
	Injurious falls and fractures	Number of falls requiring medical attention and fractures over the 12-month follow-up period.
	Physiological Profile Assessment (PPA) summary score and individual components [53, 56]	Includes five measures of physiological functioning (knee extension strength, postural sway, reaction time, lower limb proprioception and visual contrast sensitivity).
Physical activity	Incidental and Planned Exercise Questionnaire [57]	Level of physical activity relating to both basic and more demanding activities is assessed with a 10-item questionnaire.
Walking aid use	Use of walking aid	The use and type of walking aid is recorded both indoors and outdoors.
Frailty	6-point scale based on the Fried criteria [58]	Frailty is measured using five criteria: unexplained weight loss, grip strength, exhaustion, walking speed, activity level.
Pain	6-point numeric rating scale	The participant selects a whole number that best reflects the intensity of their pain.
Nutritional status	Mini Nutritional Assessment [34]	Screens for, and assesses, malnutrition in older people.
	Body mass index	Bodyweight in kilograms divided by height in metres squared.
Fall-related self-efficacy	Questions about self-rated fear of falling and balance	Participants are asked to rate their perceived balance and their fear of falling on 5-point ordinal scales
	Short version of the Falls Efficacy Scale-International [59]	Level of concern about falling during a range of activities is rated on a 4-point scale.
Mood	Five-item version of the Geriatric Depression Scale [60]	Screens mood in older people. The five-item Geriatric Depression Scale is comparable with the 15-item version in terms of psychometric properties.
Positive and negative affect	Positive and Negative Affect Scale [61]	Two 10-item scales that measure positive and negative affect.
Health-related quality of life	European Quality of Life-5 dimensions (EQ-5D-5 L) [36]	A standardised measure of health status that provides utility weights to allow calculation of quality adjusted life years (QALYs) for use in the economic evaluation [62].
	Short Form 12-item Survey (SF-12) Version 2 [63]	A 12-item questionnaire that measures functional health and well-being.
Assistance from others	Three questions about assistance received	Establishes the presence of, and reason for, assistance from agencies, family or friends.
Hospital re-admission	Number of hospital readmissions and days in hospital during the follow-up period	Ascertained via the same calendars used for falls follow-up over the first 12 months of the study, follow-up phone calls for missing calendars and contact with carers if contact is lost with the participant. At 2 and 4 years after randomisation, data linkage will be undertaken via the New South Wales Centre for Health Record Linkage (NSW CHeReL) to seek information regarding mortality and hospital admissions.
Health-system and community-service contact	Number of contacts with health and community services	Collected on a monthly basis along with the falls calendars. Inpatient hospital and emergency department contact will be assessed using data linkage via the NSW CHeReL. Data will also be used in economic analyses.
The stage of motivational readiness for change	Physical Activity Stages of Change Questionnaire [48]	The 4-item questionnaire measures the stage of readiness to change and self-efficacy to exercise.

#### Adverse events

An adverse event is defined as an incident resulting in harm to a person receiving health care [41]. The event may or may not be related to the intervention, but it occurs while the person is participating in the intervention; that is, while they are doing exercise or physical activity. For the purpose of this trial, a serious adverse event is defined as an unwanted and usually harmful outcome (e.g., fall, seizure, cardiac event). A minor adverse event will be defined as musculoskeletal soreness that interferes with activities of daily living for more for 48 hours or requires medical attention [42]. Adverse events will be monitored in the intervention group via calendars and during visits by the treating physiotherapist.

### Statistical analysis

The effect of group allocation on continuous outcomes will be assessed using linear regression models in which pre-test performance is a covariate. The number of falls per person-year will be analysed using negative binomial regression to estimate the between-group difference in fall rate [43]. The difference between the proportions of fallers in each group will be calculated using the relative risk statistic. Longitudinal analyses will be used to assess the effects on the variables measured each 3 months. Logistic regression models will be used to compare groups for dichotomous outcomes. Additional regression analyses will establish predictors for program adoption and adherence and cost-effectiveness. Sub-group analyses will be conducted using only participants with hip fracture to enable these data to be used in systematic reviews. Data will be coded to permit blinding to group allocation in the statistical analysis and the primary analyses will be conducted in accordance with the intention-to-treat principle [44]. Analyses will be conducted using the Stata software package, College Station, Texas.

### Economic evaluation

The economic evaluation will be conducted and reported in accordance with international reporting standards [45]. The economic evaluation will take the perspective of Australian health and community care funder. The cost-effectiveness analyses will include the cost of delivering the intervention (staff, training, capital costs and consumables), inpatient hospital admissions, emergency department presentations and other health and community service contact, falls rates, mobility and utility based quality of life. Incremental cost-effectiveness ratios will be calculated in terms of 1) fall prevented, 2) hospital re-admission avoided, 3) participant achieving a significant increase in mobility (one point on the SPPB [46]), and 4) QALY gained in the intervention group compared with control group, using the mean health outcomes and the mean costs in each trial arm. Bootstrapping will be used to examine the joint probability distribution of costs and outcomes; incremental cost-effectiveness planes and cost-effectiveness acceptability curves will be reported for each outcome.

### Process evaluation

A process evaluation will aim to quantify adherence, explore the participants’ experience of the intervention, and determine features associated with uptake of the intervention. This will be undertaken via calendars, verbal reports, questionnaires and a qualitative sub-study.

Adherence to the prescribed intervention will be monitored with home exercise diaries and verbal reports from participants in the intervention group. The total repetitions of home exercise performed will be divided by total repetitions prescribed. Global level of adherence will be estimated as a percentage. Participants will also be asked to identify reasons for adoption and adherence as well as non-adoption and non-adherence.

Participants’ experience of the intervention will be investigated using three questionnaires. A modified version of the Exercise Benefits/Barriers Scale [47] explores the perceived facilitators and barriers to exercise; five items measure the individual’s confidence in their ability to persist with exercising in various situations [48]; and nine questions explore the acceptability and enjoyment of the intervention.

In a subset of participants, a qualitative study will further explore the participants’ experiences of recovery from fall related fracture and participation in the study intervention program.

### Sample size calculation

A total of 350 participants (175 per group) will be recruited. The study will have 80 % power to detect as significant, at the 5 % level, a 30 % decrease in the rate of falls (i.e. an IRR of 0.70 using negative binomial regression analysis) over the 12-month study period. This sample size will provide 90 % power to detect a statistically significant between-group difference of 10 % in lower extremity continuous summary performance score [46] For these calculations, we assumed an α of 0.05, non-compliance of 15 % and a dropout rate of 15 %.

### Timeframe

Recruitment commenced in March 2010. Follow-up assessment will conclude in December 2015.

## Discussion

Many people who have suffered a lower limb fracture are left with long-term disability and increased risk of future falls, however there is no clear evidence for a successful and cost-effective program to reduce mobility-related disability and falls in fracture survivors. This randomised trial will determine whether a 12-month home exercise and falls prevention self-management program reduces mobility-related disability and falls among older men and women who live in the community following a lower limb or pelvic fracture. There will be far-reaching benefits for older people, their carers and the community if this program can assist in minimising mobility-related disability and falls in this high-risk population.

A strength of this study is that the intervention program could be delivered as part of routine care. While the program does not have as much contract with health professionals as interventions previously shown to be successful in increasing mobility after hip fracture [10, 11], we believe it is sufficiently intense to be effective, yet inexpensive enough to be implemented in real-life healthcare systems. The novel intervention combines an individualised exercise program with a self-management approach and is based on the highest level of evidence for the prescription of exercise for older people. The exercise component, the WEBB program, was designed using evidence from systematic reviews and randomised trials that have demonstrated improved strength, balance and mobility in older people. The WEBB program increases mobility in older people with frailty [49], Parkinson’s Disease [50], stroke [51], and recent hospitalisation [22]. Of concern, in our previous trail of the WEBB program in older people recently discharged from hospital the rate of falls was statistically significantly greater in the intervention group compared to the control group.^22^ The reason for increased falls is unclear, however it may be that home exercise as a single intervention may be insufficient to reduce falls in this high-risk post hospital population. The addition of the fall prevention component in the current study addresses this finding by specifically targeting fall risk and falls self-efficacy.

Additional strengths of the study are powering the study for the falls primary outcome, broad inclusion criteria, and the robust, but pragmatic, clinical trial design. Unlike most previous studies following lower limb fracture, this study is designed to detect a between-group difference in falls and is conducted over a one-year period. Importantly, the study will identify predictors of adherence to the program and will also include a comprehensive economic analysis. If effective, the intervention being examined could be readily implemented in the hospital aged care and/or community health services settings.

Falls and fractures are costly to individuals, their carers, the health system and society. Despite this cost, to our knowledge there has been no research to date examining the cost-effectiveness of intervention designed to enhance mobility and reduce falls after lower limb or pelvic fracture. In the older population, cost-benefit analysis showed the “Stepping On” program has positive net benefits and is cost saving [52]. The economic analysis conducted alongside this trial aims to establish the cost-effectiveness and cost-utility of the intervention approach, compared with that of usual care, from the perspective of the health and community care funder.

The study is not without limitations. Participants cannot be blinded to group allocation due to the complex nature of the intervention, so the potential for differential reporting of falls is a potential source of bias. However, blinding of assessors to the other primary outcome (i.e., lower extremity performance score) means this outcome is at a lower risk of bias. Also, there is no frequency-matched control group intervention, so we will be unable to determine whether social aspects of the program impact upon any difference between groups.

If this exercise and fall prevention self-management intervention is shown to reduce falls and disability in this high-risk population, there are major potential benefits to older people, their carers and the community. Avoiding falls has the potential to reduce adverse health outcomes, such as disability, hospitalisation and institutionalisation, and the associated financial costs. Enhanced mobility will likely improve functioning and result in better quality of life. If cost-effectiveness is established, this intervention will enable more efficient utilisation of health services. The findings will be disseminated in peer-reviewed journals and via professional and scientific conferences. To facilitate the adoption of the program after the results of this study are known, a full report and manual for the intervention program will be widely distributed to clinicians, health service managers and policy workers.

## Additional file

Additional file 1:
**Strategies used to maximise adherence to intervention, classified with the Behavior Change Technique Taxonomy (version 1).** ^1^(DOCX 119 kb)

## Aabbreviations

RESTORE: Recovery Exercises and STepping On afteR fracturE trial
WEBB: Weight Bearing Exercise for Better Balance
SPPB: Short Physical Performance Battery
AM-PAC: Acute Measure for Post Acute Care
LLFDI: Late Life Functioning and Disability Instrument
PPA: Physiological Profile Assessment
QALY: Quality adjusted life year
CSPS: Lower extremity continuous summary performance score
NSW CHeReL: New South Wales Centre for Health Record Linkage
IRR: Incidence rate ratio

## References

[1] Handoll HH, Sherrington C, Mak JC (2011). Interventions for improving mobility after hip fracture surgery in adults. Cochrane Database Syst Rev.

[2] Magaziner J, Hawkes W, Hebel JR (2000). Recovery from hip fracture in eight areas of function. J Gerontol A Biol Sci Med Sci.

[3] Parker M, Johansen A (2006). Hip fracture. BMJ.

[4] Ensrud KE (2013). Epidemiology of fracture risk with advancing age. J Gerontol A Biol Sci Med Sci.

[5] Auais MA, Eilayyan O, Mayo NE (2012). Extended exercise rehabilitation after hip fracture improves patients' physical function: a systematic review and meta-analysis. Phys Ther.

[6] Diong J, Allen N, Sherrington C: Structured exercise improves mobility after hip fracture: a meta-analysis with meta-regression. Br J Sports Med 2015. [Epub ahead of print] 10.1136/bjsports-2014-094465 26036676

[7] Crotty M, Unroe K, Cameron ID (2010). Rehabilitation interventions for improving physical and psychosocial functioning after hip fracture in older people. Cochrane Database Syst Rev.

[8] Sherrington C, Tiedemann A, Cameron I (2011). Physical exercise after hip fracture: an evidence overview. Eur J Phys Rehabil Med.

[9] Australian and New Zealand Hip Fracture Registry (ANZHFR) Steering Group: Australian and New Zealand Guideline for Hip Fracture Care: Improving Outcomes in Hip Fracture Management of Adults. In*.* Sydney: Australian and New Zealand Hip Fracture Registry Steering Group; 2014.

[10] Hauer K, Specht N, Schuler M, Bartsch P, Oster P (2002). Intensive physical training in geriatric patients after severe falls and hip surgery. Age Ageing.

[11] Binder EF, Brown M, Sinacore DR (2004). Effects of extended outpatient rehabilitation after hip fracture: a randomized controlled trial. JAMA.

[12] Latham NK, Harris BA, Bean JF (2014). Effect of a home-based exercise program on functional recovery following rehabilitation after hip fracture: a randomized clinical trial. JAMA.

[13] Sherrington C, Lord SR, Herbert RD (2004). A randomized controlled trial of weight-bearing versus non-weight-bearing exercise for improving physical ability after usual care for hip fracture. Arch Phys Med Rehabil.

[14] Sherrington C, Lord SR (1997). Home exercise to improve strength and walking velocity after hip fracture: a randomized controlled trial. Arch Phys Med Rehabil.

[15] World Health Organization (2001). International Classification of Functioning, Disability and Health.

[16] Orwig DL, Hochberg M, Yu-Yahiro J (2011). Delivery and outcomes of a yearlong home exercise program after hip fracture: a randomized controlled trial. Arch Intern Med.

[17] Bischoff-Ferrari HA, Dawson-Hughes B, Platz A (2010). Effect of high-dosage cholecalciferol and extended physiotherapy on complications after hip fracture: a randomized controlled trial. Arch Intern Med.

[18] Salpakoski A, Tormakangas T, Edgren J (2014). Effects of a multicomponent home-based physical rehabilitation program on mobility recovery after hip fracture: a randomized controlled trial. J Am Med Dir Assoc.

[19] Gillespie LD, Gillespie WJ, Robertson MC (2004). Interventions for preventing falls in elderly people: The Cochrane Library, Issue 3.

[20] Vogler CM, Sherrington C, Ogle SJ, Lord SR (2009). Reducing risk of falling in older people discharged from hospital: a randomized controlled trial comparing seated exercises, weight-bearing exercises, and social visits. Arch Phys Med Rehabil.

[21] Cummings SM, Cooper R, Cassie K (2009). Motivational interviewing to affect behavioral change in older adults. Res Soc Work Pract.

[22] Sherrington C, Lord SR, Vogler CM (2014). A post-hospital home exercise program improved mobility but increased falls in older people: a randomised controlled trial. PLoS One.

[23] Sherrington C, Tiedemann A, Fairhall N, Close JC, Lord SR (2011). Exercise to prevent falls in older adults: an updated meta-analysis and best practice recommendations. NSW Public Health Bull.

[24] Clemson L, Cumming RG, Kendig H (2004). The effectiveness of a community-based program for reducing the incidence of falls in the elderly: a randomized trial. J Am Geriatr Soc.

[25] Moher D, Hopewell S, Schulz KF (2010). CONSORT 2010 explanation and elaboration: updated guidelines for reporting parallel group randomised trials. BMJ.

[26] Folstein MF, Folstein SE, McHugh PR (1975). "Mini-mental state". A practical method for grading the cognitive state of patients for the clinician. J Psychiatr Res.

[27] Bandura A: Self-efficacy. The exercise of control. New York: W.H Freeman; 1997.

[28] Janis I, Mann L (1977). Decision Making: A psychological analysis of conflict, choice, and commitment.

[29] Egger G, Spark R, Lawson J (1990). Health promotion strategies and methods.

[30] Clemson L, Cumming RG, Kendig H et al: The effectiveness of a community-based program for reducing the incidence of falls in the elderly: a randomized trial. J Am Geriatr Soc 2004, 52(9):1487-1494. 10.1111/j.1532-5415.2004.52411.x 15341550

[31] Nolte S, Elsworth GR, Sinclair AJ (2007). et al. The extent and breadth of benefits from participating in chronic disease self-management courses: a national patient-reported outcomes survey. Patient Education Counseling.

[32] Clemson L, Swann M: Staying power: tips and tools for keeping you on your feet Sydney: Sydney University Press 2010.

[33] Pfeiffer E (1975). A short portable mental status questionnaire for the assessment of organic brain deficit in elderly patients. J Am Geriatr Soc.

[34] Vellas B, Villars H, Abellan G (2006). Overview of the MNA-Its history and challenges. J Nutr Health Aging.

[35] Haley SM, Coster WJ, Andres PL, Kosinski M, Ni P (2004). Score comparability of short forms and computerized adaptive testing: Simulation study with the activity measure for post-acute care. Arch Phys Med Rehabil [Haley, 2004 #57].

[36] Brooks R, Rabin R, de Charro F (2003). The measurement and valuation of health status using EQ-5D: A European perspective.

[37] Guralnik JM, Simonsick EM, Ferrucci L (1994). A short physical performance battery assessing lower extremity function: association with self-reported disability and prediction of mortality and nursing home admission. J Gerontol.

[38] Jette AM, Haley SM, Coster WJ (2002). Late life function and disability instrument: I. Development and evaluation of the disability component. J Gerontol A Biol Sci Med Sci.

[39] Onder G, Penninx BW, Lapuerta P (2002). Change in physical performance over time in older women: the Women's Health and Aging Study. J Gerontol A Biol Sci Med Sci.

[40] Gibson MJ, Andres RO, Isaacs B, Radebaugh T, Worm-Petersen J (1987). The prevention of falls in later life. Dan Med Bull.

[41] Hospital performance: adverse events treated in hospitals. Available from: http://www.aihw.gov.au/haag11-12/adverse-events/ [accessed 23 September 2015] [http://www.aihw.gov.au/haag11-12/adverse-events/]

[42] Latham NK, Anderson CS, Lee A (2003). A randomized, controlled trial of quadriceps resistance exercise and vitamin D in frail older people: the Frailty Interventions Trial in Elderly Subjects (FITNESS). J Am Geriatr Soc.

[43] Robertson MC, Campbell AJ, Herbison P (2005). Statistical analysis of efficacy in falls prevention trials. J Gerontol A Biol Sci Med Sci.

[44] Lachin JM (2000). Statistical considerations in the intent-to-treat principle. Control Clin Trials.

[45] Husereau D, Drummond M, Petrou S (2013). Consolidated Health Economic Evaluation Reporting Standards (CHEERS)--explanation and elaboration: a report of the ISPOR Health Economic Evaluation Publication Guidelines Good Reporting Practices Task Force. Value Health.

[46] Perera S, Mody SH, Woodman RC, Studenski SA (2006). Meaningful change and responsiveness in common physical performance measures in older adults. J Am Geriatr Soc.

[47] Sechrist KR, Walker SN, Pender NJ (1987). Development and psychometric evaluation of the exercise benefits/barriers scale. Res Nurs Health.

[48] Marcus BH, Selby VC, Niaura RS, Rossi JS (1992). Self-efficacy and the stages of exercise behavior change. Res Q Exerc Sport.

[49] Fairhall N, Sherrington C, Kurrle SE (2012). Effect of a multifactorial interdisciplinary intervention on mobility-related disability in frail older people: randomised controlled trial. BMC Med.

[50] Canning CG, Sherrington C, Lord SR (2015). Exercise for falls prevention in Parkinson disease: a randomized controlled trial. Neurology.

[51] Dean CM, Rissel C, Sherrington C (2012). Exercise to enhance mobility and prevent falls after stroke: the community stroke club randomized trial. Neurorehabil Neural Repair.

[52] Carande-Kulis V, Stevens JA, Florence CS, Beattie BL, Arias I (2015). A cost-benefit analysis of three older adult fall prevention interventions. J Safety Res.

[53] Barnett A, Smith B, Lord SR, Williams M, Baumand A (2003). Community-based group exercise improves balance and reduces falls in at-risk older people: a randomised controlled trial. Age Ageing.

[54] Hill KD, Bernhardt J, McGann AM, Maltese D, Berkovits D (1996). A new test of dynamic standing balance for stroke patients. Reliability, validity and comparison with healthy elderly. Physiother Can.

[55] Lord SR, Fitzpatrick RC (2001). Choice stepping reaction time: a composite measure of falls risk in older people. J Gerontol A Biol Sci Med Sci.

[56] Lord SR, Menz HB, Tiedemann A (2003). A physiological profile approach to falls risk assessment and prevention. Phys Ther.

[57] Delbaere K, Hauer K, Lord SR (2010). Evaluation of the incidental and planned activity questionnaire (IPEQ) for older people. Br J Sports Med.

[58] Fried LP, Tangen CM, Walston J (2001). Frailty in older adults: evidence for a phenotype. J Gerontol A Biol Sci Med Sci.

[59] Kempen GI, Yardley L, van Haastregt JC (2008). The Short FES-I: a shortened version of the falls efficacy scale-international to assess fear of falling. Age Ageing.

[60] Hoyl MT, Alessi CA, Harker JO (1999). Development and testing of a five-item version of the Geriatric Depression Scale. J Am Geriatr Soc.

[61] Watson D, Clark LA, Tellegen A (1988). Development and validation of brief measures of positive and negative affect: the PANAS scales. J Pers Soc Psychol.

[62] Rabin R, de Charro F (2001). EQ-5D: a measure of health status from the EuroQol Group. Ann Med.

[63] Ware J, Kosinski M, Keller SD (1996). A 12-Item Short-Form Health Survey: construction of scales and preliminary tests of reliability and validity. Med Care.

