# Supplementary material for: Exercise and fall prevention self-management to reduce mobility-related disability and falls after fall-related lower limb fracture in older people: protocol for the RESTORE (Recovery Exercises and STepping On afteR fracturE) randomised controlled trial
Source: BMC Geriatr. 2016 Feb 2;16:34. doi: 10.1186/s12877-016-0206-5 (PMC4739405; doi:10.1186/s12877-016-0206-5)
Supplement: Additional file 1: — Strategies used to maximise adherence to intervention, classified with the Behavior Change Technique Taxonomy (version 1). 1(DOCX 119 kb) [file 12877_2016_206_MOESM1_ESM.docx]

**Additional File 1.** Strategies used to maximise adherence to intervention, classified with the Behavior Change Technique Taxonomy (version 1)[^1^](#_ENREF_1)

| Strategy | | Used? | Strategy | | Used? |
| --- | --- | --- | --- | --- | --- |
| 1 | **Goals and planning** |  | **5** | **Natural consequences** |  |
| 1.1 | Goal setting (behaviour) | Yes | 5.1 | Information about health | Yes |
| 1.2 | Problem solving | Yes |  | consequences |  |
| 1.3 | Goal setting (outcome) | Yes | 5.2 | Salience of consequences | Yes |
| 1.4 | Action planning | Yes | 5.3 | Information about social and | Yes |
| 1.5 | Review behaviour goal(s) | Yes |  | environmental consequences |  |
| 1.6 | Discrepancy between | Yes | 5.4 | Monitoring of emotional | Yes |
|  | current behaviour and goal |  |  | consequences |  |
| 1.7 | Review outcome goal(s) | Yes | 5.5 | Anticipated regret | No |
| 1.8 | Behavioural contract | No | 5.6 | Information about emotional | Yes |
| 1.9 | Commitment | No |  | consequences |  |
|  |  |  |  |  |  |
| 2 | **Feedback and monitoring** |  | 6 | **Comparison of behaviour** |  |
| 2.1 | Monitoring of behaviour by | No | 6.1 | Demonstration of the behaviour | Yes |
|  | others without feedback | No | 6.2 | Social comparison | No |
| 2.2 | Feedback on behaviour | No | 6.3 | Information about others’ approval | No |
| 2.3 | Self-monitoring of | No |  |  |  |
|  | behaviour |  | 7 | **Associations** |  |
| 2.4 | Self-monitoring of outcomes | No | 7.1 | Prompts/cues | Yes |
|  | of behaviour |  | 7.2 | Cue signalling reward | No |
| 2.5 | Monitoring of outcome(s) of | Yes | 7.3 | Reduce prompts/cues | No |
|  | behaviour without feedback |  | 7.4 | Remove access to the reward | No |
| 2.6 | Biofeedback | No | 7.5 | Remove aversive stimulus | No |
| 2.7 | Feedback on outcome(s) of | No | 7.6 | Satiation | No |
|  | behaviour |  | 7.7 | Exposure | No |
| 2.8 | Monitoring of outcome(s) | Yes |  |  |  |
|  |  |  | 8 | **Repetition and substitution** |  |
| 3 | **Social support** |  | 8.1 | Behavioural practice/rehearsal | Yes |
| 3.1 | Social support (unspecified) | Yes | 8.2 | Behaviour substitution | Yes |
| 3.2 | Social support (practical) | Yes | 8.3 | Habit formation | Yes |
| 3.3 | Social support (emotional) | Yes | 8.4 | Habit reversal | Yes |
|  |  |  | 8.5 | Overcorrection | No |
| **4** | **Shaping knowledge** |  | 8.6 | Generalisation of target behaviour | Yes |
| 4.1 | Instruction on how to | Yes | 8.7 | Graded tasks | Yes |
|  | perform the behaviour |  |  |  |  |
| 4.2 | Infor about antecedents | No |  |  |  |
| 4.3 | Re-attribution | Yes |  |  |  |
| 4.4 | Behavioural experiments | No |  |  |  |
| 4.5 | Instruction on how to perform the behaviour | No |  |  |  |

| Strategy | | Used? | Strategy | | Used? |  |
| --- | --- | --- | --- | --- | --- | --- |
| **9** | **Comparison of outcomes** |  | **13** | **Identity** |  |  |
| 9.1 | Credible source | Yes | 13.1 | Identification of self as role model | Yes |  |
| 9.2 | Pros and cons | No | 13.2 | Framing/reframing | Yes | |
| 9.3 | Comparative imagining of future | Yes | 13.3 | Incompatible beliefs | No | |
|  | outcomes |  | 13.4 | Valued self-identify | No | |
|  |  |  | 13.5 | Identity associated with changed | Yes | |
| **10** | **Reward and threat** |  |  | behaviour |  | |
| 10.1 | Material incentive (behaviour) | No |  |  |  | |
| 10.2 | Material reward (behaviour) | No | **14** | **Scheduled consequences** |  | |
| 10.3 | Non-specific reward | No | 14.1 | Behaviour cost | No | |
| 10.4 | Social reward | Yes | 14.2 | Punishment | No | |
| 10.5 | Social incentive | No | 14.3 | Remove reward | No | |
| 10.6 | Non-specific incentive | No | 14.4 | Reward approximation | No | |
| 10.7 | Self-incentive | Yes | 14.5 | Rewarding completion | No | |
| 10.8 | Incentive (outcome) | No | 14.6 | Situation-specific reward | No | |
| 10.9 | Self-reward | Yes |  |  |  | |
| 10.10 | Reward (outcome) | No | **15** | **Self belief** |  | |
| 10.11 | Future punishment | No | 15.1 | Verbal persuasion about capability | Yes | |
|  |  |  | 15.2 | Mental rehearsal of successful | Yes | |
| **11** | **Regulation** |  |  | performance |  | |
| 11.1 | Pharmacological support | No | 15.3 | Focus on past success | Yes | |
| 11.2 | Reduce negative emotions | Yes | 15.4 | Self-talk | Yes | |
| 11.3 | Conserving mental resources | Yes |  |  |  | |
| 11.4 | Paradoxical instructions | No | **16** | **Covert learning** |  | |
|  |  |  | 16.1 | Imaginary punishment | No | |
| **12** | **Antecedents** |  | 16.2 | Imaginary reward | Yes | |
| 12.1 | Restructuring the physical | Yes | 16.3 | Vicarious consequences | Yes | |
|  | environment |  |  |  |  | |
| 12.2 | Restructuring the social | Yes |  |  |  | |
|  | environment |  |  |  |  | |
| 12.3 | Avoidance/reducing exposure | No |  |  |  | |
|  | to cues for the behaviour |  |  |  |  | |
| 12.4 | Distraction | No |  |  |  | |
| 12.5 | Adding objects to the | Yes |  |  |  | |
|  | environment |  |  |  |  | |
| 12.6 | Body changes | Yes |  |  |  | |

1. Michie S, Richardson M, Johnston M, et al. The behavior change technique taxonomy (v1) of 93 hierarchically clustered techniques: building an international consensus for the reporting of behavior change interventions. *Ann Behav Med* 2013;46(1):81-95.
